# Negotiating the ethical terrain in global value chains on the road towards the SDGs

**DOI:** 10.1057/s41291-025-00287-8

**Published:** 2025-02-21

**Authors:** Noemi Sinkovics, Samia Ferdous Hoque, Rudolf R. Sinkovics, Denanjalee Gunaratne

**Affiliations:** 1 Newcastle University, Business School, Newcastle upon Tyne, UK; 2 Innolab, University of Vaasa, Vaasa, Finland; 3 Manchester Metropolitan University, Manchester, UK; 4 Durham University, Business School, Durham, UK; 5 LUT University, Lappeenranta, Finland

**Keywords:** Strategic decision making, Normative ethics, GVC governance, CSR codes, Human rights, MNEs, Suppliers, Supply chain collaboration, Virtue ethics, Deontology, Consequentialism, Sustainable development goals (SDG)

## Abstract

This paper employs a pattern matching approach to explore the tensions arising from differences in the ethical dispositions of multinational enterprise (MNE) buyers and their suppliers within the Bangladeshi apparel manufacturing sector. It examines how varying ethical principles shape the development, implementation, and outcomes of corporate social responsibility (CSR) and labor standards. Our analysis resulted in the identification of four scenarios: legitimacy with friction, mitigated forced alignment, collaborative enhancement, and principled resistance. However, the scenario, principled resistance, is purely conceptual, as none of our empirical cases aligned with this category. We extend work highlighting the importance of ethical foundations for strategic decision making. This study advances the understanding of global value chain governance, particularly regarding MNEs’ contribution to the socially oriented Sustainable Development Goals. Our findings suggest that, out of the four scenarios, the combination of virtue ethics and consequentialist principles is most likely to facilitate a just transition to a more desirable state in contexts characterized by development challenges and institutional voids.

## Introduction

This paper explores the tensions between the ethical dispositions of MNE buyers and their Bangladeshi apparel manufacturers. Although MNEs are recognized for their role in promoting the UN Sustainable Development Goals (SDGs) in Asia (Eang et al., 2023; Prashantham & Birkinshaw, 2020; Van Holt et al., 2021), they also encounter criticism for engaging in unethical practices. Particularly, MNEs in the apparel industry are susceptible to this criticism. As one of Asia’s largest manufacturing sectors, the apparel industry contributes over 60% of global exports in garment, textile, and footwear, and accounts for a significant portion of total employment opportunities in the region (International Labour Organisation, 2018, 2022). MNEs have come under scrutiny for incidents such as child labor at Indian Rangan Exports, the 2013 Rana Plaza building collapse in Bangladesh, and more recent concerns about unfair payments during the COVID-19 pandemic (Antolin et al., 2021; Asmussen et al., 2023; Lee et al., 2019). In response to the criticism, MNEs have adopted diverse corporate social responsibility (CSR) measures, including private CSR standards and multi-stakeholder initiatives, aligned with global initiatives like the UN SDGs, ISO certifications, the UN Universal Declaration of Human Rights, and International Labor Standards (Anner, 2021; Ascencio et al., 2024; Castaldi et al., 2022). However, there is evidence of mixed outcomes for MNEs and their global value chain (GVC) partners (International Labour Organisation, 2018, 2022; Lund-Thomsen & Lindgreen, 2014; Soundararajan, 2023; Van Assche & Narula, 2022).

Notably, a strand of research in development studies highlights how contextual/cultural factors contribute to these mixed outcomes (e.g., Ascencio et al., 2024; Castaldi et al., 2022; Graham & Woods, 2006; Rossi, 2013; Singer & van der Ven, 2019). MNEs tend to develop CSR standards to gain efficiency in strategic GVC governance by standardizing supplier behavior worldwide, while also aiming to improve working conditions and better living standards (Anner, 2021; Clarke & Boersma, 2015; Trifković, 2017). However, there are concerns about the imperialistic approach of these standard setters potentially overpowering the voices of the poorest, most vulnerable and most marginalized GVC actors (Bae et al., 2020; Bennett, 2017; Khan & Lund-Thomsen, 2011; Lund-Thomsen, 2020). Given that the ethical ideals stemming from cultural and religious traditions in Asian societies differ significantly from those prevalent in Western societies, rigid rules pushed down the chain by MNEs may pose specific challenges to the achievement of intended outcomes (cf. Keleher & Kosko, 2019; Sinkovics et al., 2016). Suppliers may face moral dilemmas and hard choices when judging a particular dimension of a standard. Therefore, they may adopt different ways to balance conflicting ethical principles (cf. Crocker, 1991). The consequences of these tensions vary from auditing fraud, workplace abuse, gender disparities, and negative impact on lower-tier suppliers, all of which hinder the realization of the desired CSR outcomes (Bae et al., 2020; Soundararajan, 2023).

There are increasing calls to explore alternative ethical stances by considering the contextual and cultural factors from a pluralistic perspective (Anner, 2021; Castaldi et al., 2022; Kirste et al., 2024). By focusing on the ethical principles at the core of behavior, the normative approach evaluates actions that lead to harm and injustice (Karimova et al., 2023). Instead of considering CSR standards, codes, conventions, and other normalizing mechanisms as universally effective management tools across GVCs, this approach questions if they are effective in addressing sustainable development issues. It emphasizes the need to adapt these mechanisms to accommodate contextual and cultural variations (Ascencio et al., 2024; Falkenberg & Falkenberg, 2010; Singer & van der Ven, 2019).

In this paper, we focus on the human rights aspects outlined in SDG 8 Decent Work and Economic Growth including workers’ health and safety, well-being, working time, remuneration, and freedom of association (also see Castaldi et al., 2022). Traditionally, human rights have played a rather peripheral role in the conceptualization of CSR, because they are largely seen as legal or political constructs rather than moral rights (Wettstein, 2012; Wettstein et al., 2019). Thus, Wettstein (2012) proposes a conceptual integration of the two notions. This integration would mean that MNEs develop CSR standards that not only respect human rights but also take action to protect them when they are threatened and assist in their realization when they are violated. Wettstein (2012) further highlights the necessity of a set of CSR capabilities to be achieved by firms if they are to contribute to society within the boundaries of their means.

Drawing on the GVC literature, normative ethics, and human rights discourses, we argue that although MNEs possess the power to shape the rules within their value chains (Lee & Gereffi, 2015), they often lack sufficient contextual knowledge about the root causes of certain human rights issues because of their distance from local conditions (Sinkovics et al., 2015). We seek to identify the conditions under which these governance mechanisms can be both efficient and effective in realizing human rights. We selected Bangladesh as the research setting because of its status as one of the largest apparel suppliers in Asia with a history of human rights issues (Anner, 2021; International Labour Organisation, 2018).

## Conceptual background and propositions

While philosophy scholars have focused on the application and analysis of ethical theories, management scholars have remained interested in CSR (Epstein, 1987). Fischer (2004) identifies different views in the literature concerning the relationship between CSR and ethics ranging from the assertion that they are unrelated (cf. Friedman, 2007) over ethics being a dimension of CSR (cf. Carroll, 1999) to variations of how they complement each other. While some regard CSR as the manifestation of ethics at an organizational level (cf. Davidson et al., 2002), others view the two concepts as separate from each other (cf. Boatright, 2014). They confine ethics to the level of individual behavior within organizations. As a result, CSR is regarded as an organizational-level construct connected to the impact of business activity on society (Fischer, 2004).

In this paper, we take the view that management’s ethical disposition at least partially guides the way CSR is enacted in organizations. We acknowledge there are other mediating factors such as shareholder pressure, the distance between the company and the individuals affected by the actions of the company, and the quality of the relationship between organizations and the affected parties (cf. Frederiksen, 2010; Sinkovics et al., 2021). Following Chakrabarty and Bass (2015), we use a normative ethics lens to analyze organizations’ CSR actions. The rest of this section will discuss how three ethical dispositions namely, deontological, consequentialist, and virtue ethics can be expected to shape the way organizations approach CSR (see Falkenberg & Falkenberg, 2010; Karimova et al., 2023; Kirste et al., 2024). We derive several expected patterns from the literature. The purpose of expected patterns is to guide the empirical explorations in the analysis part of the paper. A pattern matching logic (Sinkovics, 2018; Yin, 2015) helps articulate how researchers expect to recognize the initial theoretical patterns in the empirical data. This qualitative analytical technique matches the expected theoretical patterns derived from the literature to observed patterns emerging from the data. The resulting matches, mismatches, or new, unexpected patterns support theorizing and theory building efforts (Bouncken et al., 2021).

### A deontological approach to CSR

Action guided by deontological ethics is determined by duty and by universal rules (Chakrabarty & Bass, 2015). The underlying idea is that certain actions need to be carried out regardless of their consequences, because they are prescribed by universal laws that should be followed by everyone (Karimova et al., 2023; Somerville & Wood, 2016). CSR actions anchored in deontology are guided by a universal set of responsibilities or duties to employees, customers, community, and society derived from national/international/religious law, national/international standards, and/or current norms of socially acceptable behavior (Chakrabarty & Bass, 2015). For example, organizations may align their CSR practices with the UN SDGs, International Labor Organization guidelines, the UN Universal Declaration of Human Rights, and ISO standards related to social sustainability (Asmussen et al., 2023; Castaldi et al., 2022). Consequently, organizations that adopt a deontological approach in formulating and executing their CSR strategies are likely to display reduced sensitivity towards contextual factors and the ramifications of such policies (Bae et al., 2020; Herkenhoff et al., 2024). For this reason, organizations often design policies without sufficiently understanding the less visible needs and circumstances (cf. Dierksmeier, 2013).

In GVCs, lead firms frequently use codes of conduct and/or standards to manage and monitor supplier activities (Jiang, 2009; Lee et al., 2019). A deontological approach to CSR action would mean that MNE buyers regard these codes/standards as a vehicle to comply with a set of universal duties across their global value chains. When operating in host countries with weaker regulations, these codes/standards can show their corporate responsibility to consumers and other relevant stakeholders inside and outside their home country (Kirste et al., 2024; Lund-Thomsen, 2020). However, since these codes/standards are often designed without extensively consulting suppliers and workers in the receiving countries, we can expect that action solely guided by deontological ethics will be less effective. (Sinkovics et al., 2016; Soundararajan, 2023; Van Assche & Narula, 2022).

Expected theoretical pattern 1a: If MNE buyers are guided by deontological principles in their design and implementation of CSR interventions (in the form of codes and standards) across their global value chains, then these actions will not be sensitive to the immediate needs and circumstances of the adopters and the intended beneficiaries.

Expected theoretical pattern 1b: If suppliers in a global value chain are guided by deontological principles, they will comply with CSR codes and standards without trying to offset their immediate negative consequences.

### A consequentialist approach to CSR

CSR action driven by consequentialist or utilitarian ethics is outcome driven (Chakrabarty & Bass, 2015). The emphasis is on maximizing happiness or at least minimizing unhappiness (Somerville & Wood, 2016). The costs and benefits of outcomes are more important for deciding for or against implementing an intervention than the costs and benefits of the intervention itself (Chakrabarty & Bass, 2015; Falkenberg & Falkenberg, 2010). Because the emphasis is on the promotion of the best possible outcome, cultural relativism, broader contextual factors, and stakeholder needs and circumstances play an important role in the design and implementation of CSR policies (Karimova et al., 2023; Singer & van der Ven, 2019). While deontological principals do not allow the deviation from universal duties and rules, consequentialists tend to adopt a ‘the end justifies the means’ attitude (Somerville & Wood, 2016).

MNEs that adopt a consequentialist approach to design and implement codes/standards in their value chains are likely to invest in anticipating the unintended consequence of their CSR interventions. As a result, they are more likely to engage in dialog with various stakeholder groups and be more open to adaptations of their codes/standards to local needs and circumstances (Anner, 2023; Lee et al., 2019). Similarly, suppliers who approach CSR from a consequentialist view focus on the consequences of fulfilling the CSR standards imposed by their MNE buyers (Hochachka, 2023). The decision regarding the extent to which an MNE’s CSR standards are implemented depends on a costs and benefits analysis (Dierksmeier, 2013; Lund-Thomsen & Lindgreen, 2014). Based on this evaluation, they then determine the boundaries within which they will implement the codes and standards or which additional measures they will take to offset the negative consequences of compliance (Castaldi et al., 2022).

Expected theoretical pattern 2a: If MNEs are guided by consequentialist principles in their design and implementation of CSR interventions (in the form of codes and standards) across their global value chains, then they will consider local needs and circumstances in the design and implementation process

Expected theoretical pattern 2b: If suppliers in a global value chain are guided by consequentialist principles, they will adapt CSR codes and standards to their circumstances or adopt measures to offset their negative consequences.

### A virtue-based approach to CSR

CSR action propelled by virtue ethics is internally motivated by individuals’ moral character (Chakrabarty & Bass, 2015; Kirste et al., 2024). Virtue ethics are often associated with transformational leadership. A leader with a long-term vision and clear ideas on how to realize those long-term goals achieves buy-in from employees and important stakeholders through the cultivation of an organizational culture conducive to change (Hochachka, 2023; Kirste et al., 2024; Wang et al., 2016). The leader and key followers are seen as moral agents. While virtue ethics are highly personal, it is also contextual in nature. This is because, without an in-depth understanding of environmental factors, the actor cannot decide the best course of action for a given situation (Falkenberg & Falkenberg, 2010; Whetstone, 2001). Organizations driven by virtue ethics emphasize “doing good” over financial gains. The organizational culture embraces incentives and rationales that justify the pursuit of right action, which is to make a valuable contribution to society (Hochachka, 2023; Wang et al., 2016).

MNEs that are guided by virtue ethics are likely to design their CSR interventions based on their own value system and moral standing. Instead of analyzing the costs and benefits of the outcome, they will focus on what they perceive as internally motivated rightful action, or put differently, the common good (Arjoon, 2000; Kirste et al., 2024). These organizations are also likely to be characterized by long-term orientation regardless of the outcomes of a cost–benefit analysis (cf. Chakrabarty & Bass, 2015). However, MNEs and suppliers that are driven by virtue ethics might prioritize their perception of stakeholder needs grounded in their internal values over assessing the cultural appropriateness of their perception.

Expected theoretical pattern 3a: If MNEs are guided by virtue-based principles for designing their CSR codes or standards, they will prioritize these over financial benefits.

Expected theoretical pattern 3a: If suppliers are guided by virtue-based principles, they will not comply with an MNE's codes if these do not align with their values, even if it is detrimental to their bottom line. 

## Methods

### Study context

The Bangladeshi garment sector serves as the setting for this study. With 5000 factories, the garments industry of Bangladesh holds the second position globally, following China, and recorded an export value of $47.38bn in the fiscal year 2022–23 (The Bangladesh Garment Manufacturers and Exporters Association, 2024). At the time of data collection, 81% of international apparel buyers preferred Bangladesh as a sourcing country because of its production capabilities and low prices (Fontana & Egels-Zandén, 2018). Nonetheless, the 4 million apparel workers experience various difficulties, including gender inequality, child labor, limited representation, and grievances resulting from their migrant backgrounds. Poor health and safety measures have resulted in intense public scrutiny, particularly after the Rana Plaza collapse in 2013. The Bangladeshi garment sector presents an ideal setting to explore the ethical aspects pertaining to the governance and compliance of CSR standards, given the ongoing labor challenges and the pressing need for societal betterment (Anner, 2021; Fontana & Egels-Zandén, 2018).

First-tier suppliers in the Bangladeshi garment sector are involved in a specific form of international outsourcing relationship with their buyers, called tacit promissory contracting. It is a type of relational governance defined as an *“outsourcing relationship whereby supplier firms are involved in recurrent discrete transactions with the same buyers since their inception or at least for a long period of time, but without the existence of any original legally binding written agreement”* (Hoque et al., 2016, p. 254). The absence of a legal agreement enhances the flexibility and the bargaining power for the MNE buyers, making termination easy and rapid, without incurring significant transaction costs. This explains why MNEs can control operations through an informal promise of a repeat purchase with no legal obligation to deliver on that promise. The higher flexibility translates into greater uncertainty for the suppliers. As a result, suppliers are usually more committed to offer reciprocity for MNEs’ promises by maintaining expected levels of performance and relationship-specific investments (Hoque et al., 2016). Under the scope of tacit promissory contracting, multinational enterprises impose the requirement on Bangladeshi suppliers to abide by their social codes, commonly known as codes of conduct, to establish standards for ethical behavior, labor practices, and overall corporate social responsibility. These social codes aim to ensure that their operations, as well as those of their suppliers, adhere to specific ethical practices (Sinkovics et al., 2016).

Since the collapse of Rana Plaza in 2013, first-tier suppliers are required to comply with the codes developed by the Accord, a 5-year legally binding agreement to maintain fire and building safety standards in Bangladeshi garment industry. Collectively formed in May 2013 by the Bangladeshi Trade Union, the International Labor Organization, civil society organizations, and 200 MNE buyers, the Accord codes were strictly enforced. During the period of investigation, the Accord compliance improved health and safety standards in 1676 factories. The 2018 Transition Accord took over from the original Accord in 2018, maintaining the objectives of the initial agreement by transferring responsibilities to a local regulatory body. The Accord represents a significant step forward in addressing the systemic safety issues in the Bangladeshi garment sector and serves as a model for improving labor conditions in global supply chains. Factories found to have safety issues were required to undertake remediation work to bring their facilities up to standard within the period of 2013–2018 (Accord, 2024). Therefore, this 5-year period provides a suitable context for exploring how MNEs have governed the enforcement of the Accord and their own code of conduct, and how their suppliers responded to those governance mechanisms.

### Study design

We adopted a multiple case study design with a pattern matching logic. Our sample comprises ten Bangladeshi garment manufacturing firms, five of which are small and five are large. We applied a purposive sampling technique to select the firms from the total population of firms in the sector. The aim was to capture similarities and differences in the ethical dispositions of these suppliers and their buyers. Table 1 provides an overview of firm characteristics.

**Table 1:** Profile of the studied firms

Firms	Year of start	Employee size	Total revenue	Ownership status	Origin of buyers	Services offered
Firm 1	2010	500	£9 million	3 investors	Netherlands	OEM (LSN)
Firm 2	2009	600	£13 million	Family	Netherlands, Belgium, and Spain	OEM (LSN)
Firm 3	2010	550	£6 million	2 investors	UK and Italy	OEM (LSN)
Firm 4	1988	650	£14 million	Family	Germany, Spain, UK	OEM (LSN)
Firm 5	2007	80	£0.07 million	Single	Turkey, China	CMT
Firm 6	1989	1500	£29 million	Single	Sweden, Japan, Australia, Germany	OEM (VI)
Firm 7	1989	2200	£37 million	Single	USA and UK	OEM (VI)
Firm 8	1987	9000	£35 million	3 investors	Sweden and UK	OEM (VI)
Firm 9	1985	12,000	£450 million	Public Ltd	USA, UK, Spain, Italy	OEM (VI), ODM, OBM
Firm 10	1994	11,000	£380 million	Family	USA, Sweden, UK, Germany	OEM (VI), ODM, OBM

Key: Service offered: *CMT* cut, make, and trim; *OEM (LSN)* Original equipment manufacturer with own external supplier network, *OEM (VI)* Original equipment manufacturer with vertically integrated raw material production units, *ODM* original design manufacturer, *OBM* Original brand manufacturer (typology adapted from Gereffi and Frederick (2010))

We collected data in two phases: initially in 2014, right after the Accord was established, and later in 2017, just before the Accord was dissolved. This two-phase data collection enabled us to analyze the changes that occurred during the Accord agreement period, a time when compliance with CSR codes was a top priority for MNE buyers. The first stage of data collection involved fifteen one-hour interviews with managers/owners of the ten selected Bangladeshi garment manufacturers. Interviews provided information about their compliance status with MNE’s code of conduct and the Accord standards, as well as any other proactive CSR activities they might have undertaken. The interview guide also encompassed inquiries regarding their connections with their buyers, including the existence of a contract, the duration of the relationship, the ordering process, the execution and completion of transactions, the exchange of knowledge and information, the methods of contacting buyers, and influential factors in maintaining recurring relationships. Follow-up interviews were conducted in 2017 to obtain more specific insights regarding the ethical principles guiding their reactive and proactive CSR initiatives, the tensions encountered in implementing those actions, and their corresponding solutions. We further asked about the consequences of their initiatives on themselves and their workers. The interviews were complemented with corporate presentations by owners and compliance managers, factory visits, and participant observation to understand their position to develop CSR capabilities.

Data about MNEs were collected from company websites, financial and sustainability reports, and newspaper articles. The statements in these annual reports and websites represent the organizations’ signaled ethical values. Subsequently, we can match these signals to their observed strategic actions. The accounting literature often relies on publicly available data to analyze a company’s intangibles for investment, credit, and related decisions (Cañibano et al., 2000). Sustainability and social reports that undergo third-party audits and assurance are considered reasonably reliable secondary data that document the measures taken by MNEs to ensure accountability in sustainability (Perego & Kolk, 2012). Thus, we used these secondary data sources as proxy for MNEs’ CSR policy and ethical disposition. In addition, we made inquiries about the suppliers’ perceptions of the buyer organization’s CSR initiatives.

### Analytical approach

We performed the data analysis in several steps. To begin, we relied on the broad expected patterns from Sect. “Conceptual background and propositions” to determine the ethical dispositions of MNE buyers and suppliers. This categorization allowed us to identify different scenarios based on the combinations of ethical dispositions. The next step involved the construction of an initial template based on the dimensions derived from the MNEs’ code of conduct and the Accord standards. We recorded the status of compliance with those standards, the contextual and cultural constraints experienced by the firm in implementing those standards, and the subsequent consequence for workers (cf. Oya et al., 2018). The implications of code compliance (or non-compliance) for workers were determined with the help of the Human Rights Measurement Framework developed by Equality and Human Rights Commission. The framework has six domains, which reflect the things or areas in life that are important to people and enable them to flourish: Education, Work, Living standards, Health, Justice and personal security, and Participation (EHRC, 2017). Table 2 presents a summary.

**Table 2:** CSR compliance and consequences for workers

CSR dimensions	MNEs in scenario 1 and 2	Scenario 1		Scenario 2		Scenario 3	
Codes		Supplier firm: case 4,5	Supplier firm: case 9, 10	Supplier firm: case 1, 2, 3	Supplier firm: case 6,7	MNE associated with case 8	Supplier firm: case 8
**Conditions of service and employment**	MNE is strict on having majority workers as full-time contractual ones	Formal employment contract for workers	Formal employment contract for workers	Part-time/seasonal workers (20%) and full-time (80%); No formal employment contract for workers	Full-time (98%) and part-time (2%); Service contract provided	MNE is not strict on the ratio of full-time vs. part-time workers	Full-time (95%) and part-time (2%); Service contract provided
Consequences for workers		Personal security: Increased job security, legal protection of rights and benefits (all firms)		Personal security: Increased job security, legal protection of rights and benefits (all firms)		Work arrangements: Many workers prefer flexible arrangement as they can more easily switch jobs	
**Maternity benefits**	MNE is strict on providing maternity benefits	Maternity benefits provided Suppliers tend to avoid hiring married workers (only 5%)	Maternity benefits provided	Suppliers tend to avoid hiring married workers (only 5%)	Maternity benefits provided Married and unmarried workers get equal opportunity to get recruited	MNE shares the additional cost of offering maternity benefits	Maternity benefits provided Married and unmarried workers get equal opportunity to get recruited
Consequences for workers		Living standard: Increased financial security during maternity period and better control on family planning (in firms 9, 10) Living standard: Married women face difficulty in getting a job (in firms 4,5)		Living standard: Increased financial security during maternity period and better control on family planning (in firms 6, 7) Living standard: Married women face difficulty in getting job (in firms 1,2,3)		Living standard: Increased financial security during maternity period and better control on family planning	
**Health, safety, and hygiene**	MNE strictly follows the Accord and their own codes on health and hygiene	Meet requirements for fire safety, ventilation, and space, clean drinking water, toilet facilities	Meet requirements for fire safety, ventilation, and space, clean drinking water, toilet facilities	Complied with 60% of requirements Advised to increase floor space, number of exhaust fans, and overall ventilation Advised to provide additional light fixtures to increase illumination	Meet requirements for fire safety, ventilation, and space, clean drinking water, toilet facilities, air coolers	MNE strictly follows their own codes on health and hygiene; however, provides financial support and training for implementation	Meet requirements for fire safety, ventilation, and space, clean drinking water Exhaust pipe with high suction capacity for controlling heat and fume on the factory floor
Consequences for workers		Health: Improved working environment (all firms) Safety: limited risk of fire (all firms) Employment implications: Increased cost of compliance compensated through automation and downsizing of unskilled workers (in firms 4, 5)		Health: Improved working environment in terms of cleanliness and spaciousness (all firms) Safety: limited risk of fire (all firms) Employment implications: Increased cost of compliance compensated through automation and downsizing of unskilled workers (in firms 1,2,3)		Health: Improved working environment in terms of cleanliness and spaciousness (all firms) Safety: limited risk of fire Employment implications: Increased cost of compliance was not passed on to workers	
**Welfare**	MNE strictly follows own codes on medical, childcare, and dining facilities	Complies with medical facility requirements Childcare and dining facility	Complies with medical facility requirements Childcare and dining facility	Complies with medical facility requirements No childcare and dining facilities	Medical unit serves entire campus Childcare unit with pre-school education facility Canteen for workers and other staff	MNE provides financial support for implementing these measures	Medical unit serves entire campus Childcare unit with pre-school education facility Canteen for workers and other staff
Consequences for workers		Health: Workers get basic healthcare when there is an accidents or minor sickness (all firms) Employment implications: Increased cost of compliance compensated through automation and downsizing of unskilled workers (in firms 4, 5)		Health and standard of living: These facilities improve overall standard of living for workers (in firms 6,7) Participation: Workers are more motivated to work productively (in firms 6,7) Employment implications: Increased cost of compliance compensated through redirecting funds from other initiatives (in firms 1,2,3)		Health and standard of living: These facilities improve overall standard of living of the workers Participation: Workers are more motivated to work productively Employment implications: Increased cost of compliance was not passed on to workers	
**Working hours and leave**	MNE strict on compliance with legal overtime limit	No overtime	Meets the legal overtime limit	Overtime applied when needed but overall, there is a reduction in overtime	Generally, meets legal maximum but sometimes goes beyond	MNE not strict on overtime hours	Generally, meets legal maximum but sometimes goes beyond
Consequences for workers		Standard of living: Increased leisure time to rest and relax (all firms), but reduced monthly income (all firms)		Standard of living: Increased leisure time to rest and relax (all firms), but reduced monthly income (all firms)		Standard of living: Increased leisure time to rest and relax (all firms), but reduced monthly income	
**Wages and payment**	Expects compliance with national minimum wage	Compliance with the national minimum wage rate for full-time and part-time workers	Compliance with the national minimum wage rate for full-time and part-time workers	Compliance with the national minimum wage rate for full-time and part-time workers	Provides living wage beyond national minimum and other employee benefits	MNE expects compliance with the national minimum wage regulation	Provides living wage beyond national minimum and other employee benefits
Consequences for workers		Personal security: Legal protection to receive minimum wage (all firms) Standard of living: Minimum wage does not equal living wage due to rising cost of housing, food and other basic facilities in the absence of market regulation (all firms)		Personal security: Legal protection to receive minimum wage (all firms) Standard of living: Minimum wage does not equal living wage due to rising cost of housing, food, and other basic facilities in the absence of market regulation (in firms 1,2,3), however, better standard of living due to above average salary (in firms 6,7)		Standard of living: workers enjoy better standard of living due to above average salary	
**Trade union and industrial relations**	Pressure on suppliers to allow trade union participation	Workers can join the trade union	Workers can join the trade union	Workers are restricted from trade union participation	Workers are discouraged from trade union participation Instead, workers’ participatory committee (WPC) is created: elected representatives to liaise with management Continuous dialog between middle-management and workers to identify and solve problems in their work and private lives Research team to identify issue in workers’ work and private lives	MNE does not pressure supplier to allow trade union participation MNE appointed external consultancy firm to liaise with workers and provide them with training on empowerment	Workers are discouraged from participating in trade unions Instead, workers’ participatory committee (WPC) is created: elected representatives to liaise with management Workers collaborate with MNE appointed consultancy firm to resolve issues and to receive training
Consequences for workers		Workers can join trade unions and thus be part of external and larger communities (all firms), potential risk of being drawn into activities that could escalate to violence		Participation: Resolution of issues through communication with management (all firms) Justice and personal security: Management views WPC as a way to protect workers from being exploited by the union leaders (all firms)		Participation: Management views WPC as a way to protect workers from being exploited by the union leaders	
**No discrimination**	Expectation to maintain a balanced ratio of male and female workers at every level and department	Female supervisors on some floors	Female supervisors on some floors	Workers: Female (80% as helpers and operators), male (cutting and packaging 20%) Supervisors: Male (100%) Management: Male (10%); female (90%)	Workers: Male 60% and female 40% Supervisors: Male 100%	MNE appointed external consultancy firm to train female workers on how they can express their voice, tackle tricky situations and train male workers on how they should respect female colleagues and accept female leadership	Workers: Male 30% and female 70% Supervisors: both male and female
Consequences for workers		Participation: male workers reluctant to listen to female supervisors (all firms) Opportunity for female workers to get promoted to supervisory level (all firms)		Participation: Male supervisors/managers are seen as more effective to maintain control and harmony on the factory floor (all firms) Limited opportunity for female workers to get promoted to supervisory level (all firms)		Participation: female leaders perform with confidence and males respect them Opportunity for female workers to get promoted to supervisory level	
**General building requirement**	Shared factory setting strictly prohibited	Rented the entire building to avoid shared setting	Established independent factory building on own land	Still in a rental shared building	Independent campus with separate buildings for each unit	MNE provides financial support to help with this requirement	Established independent factory building on their own land
Consequences for workers		Personal security: Very low risk of building collapse or other accidents Work: The beautiful campus increases the motivation of the workers Standard of living: Increased cost of compliance compensated through automation and downsizing of unskilled workers (in firms 4, 5,)		Personal security: Reduced risk of building collapse or other accidents (Firms 6,7) Work: The beautiful campus increases the motivation of the workers (Firms 6,7)		Personal security: Reduced risk of building collapse or other accidents Work: The beautiful campus increases the motivation of the workers Standard of living: Increased cost of compliance was not passed on to workers	
**Proactive CSR activities**	No incentive for suppliers to engage in additional, proactive CSR activities	No additional actions	No additional actions	Free lunch for workers Festive bonus to workers Charity in the local community during festive times and for schools, hospitals, etc. Regular donation for local orphanage Separate prayer room and prayer break for workers	Free lunch for workers Opened 14 primary schools in the community providing free education (60% of workers’ children and 40% community) Provident fund, annual leave, maternity benefits, health and education loan Free treatment and medicine for workers in the medical unit; subsidized treatment facility for workers’ family Free transportation for workers and staff Introduced parenting resources Job opportunities for the family members of workers to increase their commitment and family income (30% of workers have relatives working in Firms 6, 7)	MNE provides extra value for supplier’s proactive CSR initiatives such as, paying above market price, long-term contract, and financial support for innovative CSR initiatives	Started providing free medicine for workers and their children Organize health camp whereby free primary care is given to workers and their immediate family members Free cooked lunch Pre-school educational facility in the childcare unit by qualified teachers Other financial and non-financial support to workers in time of need Provide interest-free loan to workers to ensure their high morale and better life Gift for newborn baby Free transport facility Planning to start provident fund from 2015
Consequences for workers		Health, education, living standard: The social constraints faced by workers are not addressed (all firms)		Standard of living: Workers get complete nutrition, privacy to practice religion, savings on lunch expenses, financial assistance during festive times (all firms) Health, and education: improved the education, health, and overall quality of life of disadvantaged in local community (all firms)		Health, education and living standard: The social constraints faced by workers are targeted by the initiatives	

**Fig. 1:**
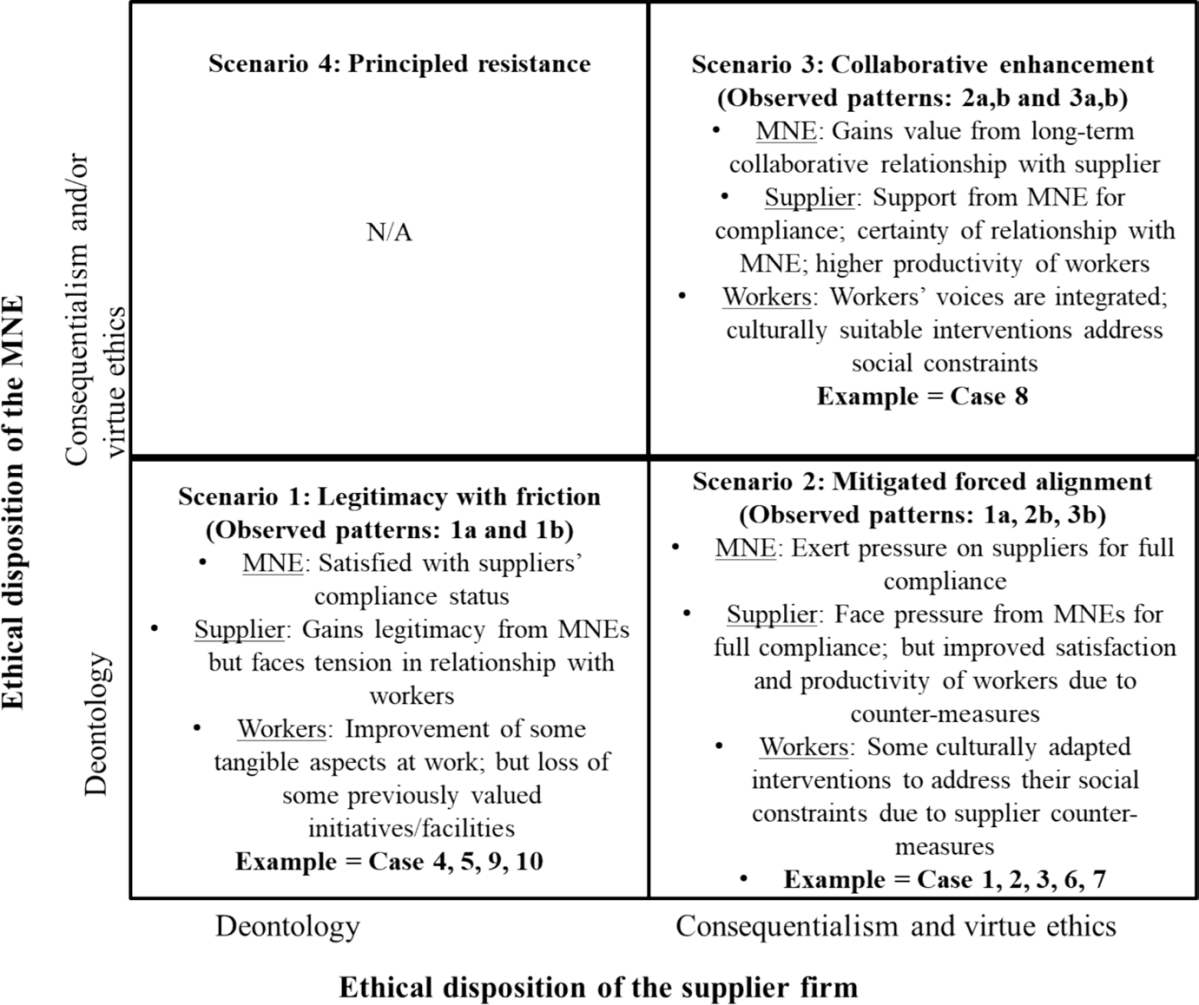
Ethical dispositions in MNE–supplier relations: A scenario matrix

Subsequently, we performed pattern matches. We recorded the outcomes in Table 3. To arrive at the pattern matches, we used the initial expected patterns and operationalized them in more detail (Sinkovics, 2018; Yin, 2015; Yin & Moore, 1988). To do so, we drew inspiration from Chakrabarty and Bass (2015). The coding and analysis underlying the pattern matches were performed in NVivo. The last step involved the creation of Fig. 1 that summarizes the main insights in a two-by-two matrix to reflect the four scenarios.

**Table 3:** Pattern match of expected patterns to observed patterns

Operationalization of dimension	Theoretical pattern	Expected pattern	Observed pattern	Implications
Patterns 1a and 1b derived from Deontological ethics				
Focus on action vs. consequence	Action driven	**MNEs**: Focus on universal duties to protect human rights in countries where they operate **Suppliers**: Focus on implementation of MNEs’ universal standards	**MNEs**: the buyers of Firms 1, 2, 3, 4, 5, 6, 7, 9, and 10 require suppliers to have at least the Accord amber and 60–70% compliance with their own codes, eventually leading to 100% compliance **Suppliers**: Only firms 9 and 10 have 100% compliance; Firms 4, 5 are in the process of full compliance	Pattern 1a observed in MNE buyers of Firms 1, 2, 3, 4, 5, 6, 7, 9, and 10 and pattern 1b observed for supplier firms 4, 5, 9, and 10
Stakeholders need	Not important	**MNEs**: Design CSR codes with no consideration of stakeholders’ context-specific need **Suppliers**: Willingly follow MNE codes with no consideration of stakeholders’ context-specific need	**MNEs**: the buyers of Firms 1, 2, 3, 4, 5, 6, 7, 9, and 10 have no mechanism to integrate stakeholders’ voice in code development and implementation **Suppliers**: While Firms 4, 5, 9, and 10 understand stakeholder needs, they must ignore them to comply with MNEs’ codes	Observation based on pattern match: *Not all suppliers follow MNE expectations when these do not suit the local context. Supplier firm leaders understand stakeholder needs and what is culturally appropriate. They do not always willingly pursue 100% compliance; they do so out of obligation to maintain relationships with buyers when they do not have any wriggle room to oppose. As they are embedded in the local context, they see the downside of following deontological principles. Where the MNE buyer is attempting to enforce their deontological principles, there is a ****tension**** in the buyer–supplier relationship*
Cultural relativism	Not important	**MNEs**: No consideration of cultural factors in designing codes **Suppliers**: Implement MNE codes without attempt to adapting to cultural context	**MNEs**: the buyers of Firms 1, 2, 3, 4, 5, 6, 7, 9, and 10 do not consider cultural appropriateness in designing codes **Suppliers**: Firms 4, 5, 9, and 10 realize that some codes are culturally inappropriate, despite being required to achieve 100% compliance	
Value orientation	Outward oriented	**MNEs**: Demonstrate dutiful behavior towards stakeholders in developing countries based on universal principles **Suppliers**: Demonstrate dutiful behavior by implementation of MNE codes and adapt their internal values accordingly	**MNEs**: the buyers of Firms 1, 2, 3, 4, 5, 6, 7, 9, and 10 follow universal principles of human rights and they design internal values based on those principles **Suppliers**: Firms 4, 5, 9, and 10 follow MNEs’ codes even if their internal values suggest otherwise	
Role of leader	Less important	**MNEs**: CSR actions are driven by MNEs universal duties of care rather than the leader’s ideology **Suppliers**: CSR actions are externally driven to reflect duties of care rather than being influenced by the leader	**MNEs**: The buyers of Firms 1, 2, 3, 4, 5, 6, 7, 9, and 10 follow the same universal codes for all suppliers worldwide, despite regional or local differences **Suppliers**: Firms 4, 5, 9, and 10 follow MNEs’ codes despite those conflicting with their leaders’ personal ideology	
Patterns 2a and 2b derived from Consequentialist ethics				
Focus on action vs consequence	Consequence driven	**MNEs**: Focus on a beneficial outcome for the suppliers and their stakeholders **Suppliers**: Decision regarding the extant of implementation of MNEs’ CSR standards is likely to depend on the analysis of costs and benefits of doing so	**MNEs**: Only the buyer of Firm 8 showed some degree of willingness to adapt codes for stakeholders’ benefit **Suppliers**: Firms 1, 2, 3, 6, 7, and 8 have complied with most MNE codes, while adapting the rest to optimize benefits for stakeholders	Pattern 2a observed in MNE buyer of Firm 8 and pattern 2b observed in Firms 1, 2, 3, 6, 7, and 8
Stakeholders need	Very important	**MNEs**: Design CSR codes in consideration of stakeholders’ context-specific needs **Suppliers**: Decision regarding the extent of implementation of MNEs’ CSR standards is likely based on an analysis of stakeholders’ needs	**MNEs**: Only the buyer of Firm 8 showed some degree of consideration for stakeholders’ context-specific needs **Suppliers**: Firms 1, 2, 3, 6, 7, and 8 prioritize stakeholders’ context-specific needs wherever possible, even if those go against MNE codes	Observation based on pattern match: *The ethical dispositions of the buyers of Firms 1, 2, 3, 6, and 7 do not match those of their suppliers. While these buyers follow deontology, their suppliers follow and implement codes based on consequentialism. This ****creates tension****, causing suppliers to doubt their ability to continue prioritizing stakeholders’ needs and cultural relativism*
Cultural relativism	Very important	**MNEs**: Design CSR actions based on cultural appropriateness **Suppliers**: Decision regarding the extant of implementation of MNEs’ CSR standards is likely to depend on cultural appropriateness	**MNEs**: Only the buyer of Firm 8 showed some degree of consideration for cultural relativism **Suppliers**: Firms 1, 2, 3, 6, 7, and 8 adapt the codes for cultural appropriateness wherever possible, even if those go against MNE codes	
Value orientation	Outward oriented	**MNEs**: CSR actions are to produce greatest good for suppliers and their stakeholders even if firms own values do not match Suppliers: CSR actions are to produce greatest**Suppliers**good for stakeholders even if firms own values don’t match	**MNEs**: Only the buyer of Firm 8 value stakeholders’ need, which also resonates with their internal values **Suppliers**: Firms 1, 2, 3, 6, 7, and 8 prioritize creating the greatest good for stakeholders which also resonate with their internal values	
Role of leader	Less important	**MNEs**: CSR actions are outcome driven even if the leaders’ ideology does not match **Suppliers**: CSR actions are outcome driven even if the leaders’ ideology does not match	**MNEs**: Only the buyer of Firm 8 design codes to generate optimum outcome for stakeholders, which also resonates their leaders’ values **Suppliers**: Firmd 1, 2, 3, 6, 7, and 8 prioritize creating the greatest good for stakeholders which also resonate their leaders’ personal values	
Expected patterns 3a and 3b derived from Virtue ethics				
Focus on action vs. consequence	Action driven	**MNEs**: Design CSR actions based on internal values rather than any universal rule, law, or outcome **Suppliers**: Decision regarding the extant of implementation of MNEs’ CSR standards depends on firm leader’s internal values	**MNEs**: Only the buyer of Firm 8 demonstrates some actions driven by strong internal values that are not influenced by universal rules or cost–benefit analysis **Suppliers**: Firm 8’s CSR actions driven by their organizational or leaders’ personal values rather than universal rule or cost–benefit analysis	Patterns 3a and 3b were only observed in Firm 8 and their buyer
Stakeholder needs	Moderately important	**MNEs**: Focus on internal moral values more than immediate stakeholder need **Suppliers**: Focus on internal moral values more than immediate stakeholder need	**MNEs**: Only the buyer of Firm 8 considers immediate stakeholder need, which also resonates with their internal values **Suppliers**: Firm 8 considers stakeholder need, which also resonates with their internal values	Observation based on pattern match: *Both Firm 8 and their MNE buyer consider stakeholder needs and cultural appropriateness because their internal moral values also suggest doing so. Because of the alignment of their internal values, there is ****limited tension**** in their relationship*
Cultural relativism	Moderately important	**MNEs**: Focus on internal moral values rather than cultural appropriateness **Suppliers**: Focus on internal moral values rather than cultural appropriateness	**MNEs**: Only the buyer of Firm 8’s internal moral values suggest considering cultural relativism **Suppliers**: Firm 8’s internal moral values suggest considering cultural relativism	
Value orientation	Both inward and outward oriented	**MNEs**: CSR actions and firms’ internal moral values match **Suppliers**: CSR actions and firms’ internal moral values match	**MNEs**: The buyer of Firm 8’s CSR actions are in line with their internal values **Suppliers**: Firm 8’s CSR actions are in line with their internal values	
Role of leader	Very important	**MNEs**: The moral values of the leader drive CSR actions to a large extent **Suppliers**: The moral values of the leader drive CSR actions to a large extent	**MNEs**: The buyers of Firm 8’s CSR actions are primarily driven by the moral values of their leader, which emphasize considering stakeholder needs and cultural relativism **Suppliers**: Firm 8’s CSR actions are largely driven by their leaders’ personal and family ideology

## Findings

### Scenario 1: legitimacy with friction

We labeled the first scenario *legitimacy with friction*. Supplier firms 4, 5, 9, and 10 reported that their MNE buyers are inflexible about the dimensions outlined in the codes and standards. The following quotes from the MNEs’ corporate websites and CSR reports exemplify their stance and corroborate the suppliers’ perceptions:

“All our suppliers and manufacturers worldwide are required to follow our code of conduct, which applies the highest standards for the protection of human rights and the promotion of international labor rights, health and safety, and environmental aspects. The Code applies not only to suppliers but all their facilities down to the last production unit. Through our traceability systems, we know exactly how our products are made and where they come from.” (CSR Report, MNE buyer of Firm 5)

“We are a signatory of the Accord on Fire and Building Safety in Bangladesh, which aims for sustainable improvements in working conditions in the Bangladesh garment industry. However, we will not compromise on either quality or compliance with codes and maintain a competitive stance on price. We only source from suppliers who meet our standards or have committed to achieving our standards within an agreed timescale.” (Sustainability Report, MNE buyer of Firm 9)

Referring to their MNE buyers’ stance on CSR codes, all four supplier firms saw value in their universal approach to standard design and implementation. Simultaneously, they emphasized that a dialog would have yielded more culturally acceptable solutions for their workers.

“I believe full compliance significantly improves working conditions. I respect their [MNE buyers’] ideology because I have seen the catastrophe resulting from non-compliance, as in the case of Rana Plaza. Yet, I believe there should be some room for adaptation and consultation. We can be guided by them [MNEs] but not dictated.” (Owner, Firm 5)

“Our buyers have the expertise in designing the highest standard of CSR codes. From their worldwide operations and research activities, they have developed the skills to govern these codes. It is for our best. I believe the entire industry is much more professional and transparent now, which gives us a competitive advantage over our competitors like China and India. However, I believe some adaptations are necessary in every country to ensure better acceptance of the codes.” (Managing Director of Firm 10)

Table 2 summarizes the different MNE requirements, the suppliers’ response to these requirements, and the consequences of the responses for workers. Requirements include the provision of formal employment contracts, specific quotas for full-time and part-time workers, maternity benefits, minimum wage, healthcare, and childcare facilities. Implementing these codes resulted in higher job security and better legal protection pertaining to minimum wage, punctual payment, and various other rights and benefits. Female workers experience increased financial stability during the period of maternity and improved ability to manage family planning. Further requirements stipulated compliance with health, hygiene, and safety codes, ensuring fire safety measures, ventilation, more space, access to clean drinking water, and sufficient toilet facilities. Suppliers were also obligated to adhere to the building safety standards outlined by the Accord, which include electrical safety, structural robustness, and avoiding shared rental buildings. Compliance with these codes improved the working environment and reduced the risk of incidents such as fires and building collapses. MNE buyers in this scenario also exerted pressure on supplier firms to support their workers’ freedom of association and allow them to join trade unions.

Overall, the strict compliance with the codes achieved benefits for the workers. However, the interviews revealed that these measures also created some unintended consequences. For example, respondents from firms 4 and 5 admitted that the provision of additional maternity and childcare benefits influenced their decision to hire unmarried individuals, consequently creating barriers for married women to pursue job opportunities within the sector. Further, the smaller firms in this scenario had limited access to bank loans and other institutional support. As a result, to compensate for the cost of compliance, they were forced to replace unskilled workers with automated machines.

“We could have given the opportunity to helpers to become more skilled by offering them more on-the-job training. However, doing so would have required more money and time, which we did not have at that time. We had to act fast to impress our buyers so that they continue taking orders from us. There is no legal commitment from the buyers to continue the relationship, which means if we had not acted fast, they could have switched. Therefore, for the sake of business survival and the welfare of most of our workers, we had to take a hard decision.” (Owner, Firm 5)

The high level of labor migration in the industry further increased the financial risk for the smaller firms. The absence of public or private institutions for training garment workers made it even more difficult for small firms to launch training initiatives at their own expense. Therefore, while the improved working environment enhanced the health and physical security of workers who remained, there was no protection for unskilled workers who lost their jobs and struggled to find new employment. Other unintended consequences stemmed from firms’ limited control over contextual challenges. For instance, despite complying with minimum wage regulations, they could not protect workers’ rights to maintain a decent standard of living. This is partly because the new minimum wage set by the government was below the living wage, and partly because firms have limited resources and thus cannot keep up with compensating price increases. Thus, the lack of regulatory control over food prices, rent, and the prices of other necessities eroded any increase in workers’ wages. The imposed cap on overtime hours further reduced the monthly income of workers. Further, paying a living wage to workers does not seem to lead to an increase in the willingness of buyers to pay higher prices.

The two large firms (Firms 9 and 10) in this scenario had more resources. For them, the recommended modifications following the initial audit were minor, as they had the resources to design the factory buildings with the workers’ safety and comfort in mind. Both firms took proactive measures to introduce several facilities, including childcare, medical, clean drinking water, and canteen facilities, even before feeling the pressure to comply with the Accord codes. In addition, they underwent continuous upgrading to comply with the changing demands of buyers. The interviewees from Firms 9 and 10 still reported mixed results for workers. Although they safeguarded their workers from potential negative repercussions, such as job loss, they were forced to discontinue certain facilities that the workers prioritized over those mandated by the Accord. For instance, Firm 9 reported that they used to give free clothes to workers and their immediate family members during the Eid religious festival, which they stopped a year before our data collection. As most workers adhere to the Islamic faith, they showed a strong preference for this benefit, as it effectively lessened their financial burden during the festive season. The head of the labor management division commented:

“Our workers do not need a soap dispenser, toilet tissues and commode seats in the toilet; they were rather happy with bar soap, a gamcha [traditional cloth serving the purpose of towels] and traditional pan system toilets... We have made a new factory with all modern amenities to provide a comforting and safe environment to the workers; however, we know how much they [workers] miss the free clothes and bonuses that we used to give during Eid times.” (Head of Labor Division, Firm 9)

Similarly, the owner of Firm 10 mentioned how their workers objected to the decision to stop providing a free lunch. They prioritized the lunch facility over other newly added facilities, some of which they hardly used, such as the childcare facility. In his own words:

“Previously they used to get meat/fish, vegetable, lentil and rice as free lunch; whereas now they can only afford to have potato curry and rice for lunch. This change has been a sudden pressure on workers’ financial arrangements. Of course, they appreciate the new compliant factory; however, I believe they would have never agreed to give away the lunch facility for those changes.” (Owner, Firm 10)

Therefore, while the suppliers in this scenario did not question the universal value of the measures, they questioned the lack of dialog. Their contextual and cultural knowledge could have helped to mitigate the unintended consequences of compliance for workers.

### Scenario 2: mitigated forced alignment

Similarly to the MNE buyers in scenario 1, buyers in scenario 2, labeled *mitigated forced alignment*, also show a firm commitment to enforcing their universal codes. The MNE buyer of Firm 6 has a “Zero-tolerance” policy. They make use of a rigorous auditing process to monitor suppliers’ compliance levels and assign ratings from A to E based on specific assessment criteria for each element of their Supplier Code of Conduct. The supplier management report on their website clearly states:

“It is our policy not to place production orders with E-rated suppliers—although we work closely with them to address these issues and improve their rating over time, so they are able to receive new orders in the future. New suppliers and production units must be able to demonstrate that they meet our sustainability criteria, and if needed, make improvements before they can start working with us...To continually improve our auditing process and drive the right behavior, we update our audit protocols every year to raise the bar on our standards over time.” (Sustainability Report, MNE buyer of Firm 6)

Several prominent buyers of Firm 6 and Firm 7 have already streamlined their supply chain to enhance monitoring capabilities. While many of them work in partnership with tier 1 suppliers to improve their capacity, they expect complete compliance with codes in return. Full compliance often is a prerequisite to start any new relationship or maintain an existing one. The key buyers of Firms 1, 2, and 3 have similar supplier management policies and practices. Most of them work with the Accord to audit and rate them. They have their own voluntary code of conduct. The buyers require suppliers to have at least the Accord Amber grade and 60–70% compliance with their own codes to continue the relationship. The MNE buyers in our sample did not display a willingness to consider tailoring their codes to accommodate the specific demands of local contexts.

“In 21 of our sourcing countries, we implement our own code of conduct. Our suppliers in all 21 countries are obligated to comply with these universal requirements.” (Social Report, MNE buyer of Firm 6)

“All 3000 of our suppliers must adhere to our Global Sourcing Principles, which cover working conditions and workers’ rights.” (Annual Report, MNE buyer of Firm 7)

“Goods sold by us are currently manufactured in over 1000 factories through 688 suppliers...We provide tools such as our Code of Conduct Guidebook to our suppliers worldwide to support and accelerate the resolution of problems identified during audits. We have a traffic light system grading system. A red light would signal severe non-compliance with the codes. If a factory audit is graded red, we will not work with this factory until the issues have been resolved. (Annual Report, MNE buyer of Firm 3)

When non-compliance occurs, buyers rarely conducted a thorough inquiry to uncover the root cause, nor did they consider geographical and cultural differences. Based on the secondary data, these MNEs seemed to be guided by deontological principles. Similarly to scenario 1, the voice of suppliers was rarely acknowledged in the establishment or enforcement of codes, a sentiment echoed by the owners/managers of supplier firms.

“We know very well about our culture and our workers’ problems, which the buyers are unaware of. However, there is no way to communicate those to them.” (Chairman, Firm 1)

“We know several codes are inappropriate and will not produce the expected result. However, we cannot point that out to the buyers because they are not ready to listen.” (Compliance Manager, Firm 6)

The analysis shows that Firms 1, 2, 3, 6, and 7 implemented or were in the process of implementing changes suggested by the buyers. However, in contrast to suppliers in scenario 1, these firms also launched some countermeasures to buffer against some negative outcomes of code compliance. Thus, we classified these suppliers as guided by consequentialist principles. They clearly conducted a cost–benefit analysis to determine the extent to which they can disregard their MNE buyer’s CSR standards. In this process, the suppliers focused on the outcomes of code compliance instead of strictly following the codes.

For example, one MNE’s codes required suppliers not to exceed the threshold of 5% for part-time workers and to issue employment contracts to all workers. However, Firms 1, 2, and 3 employ 20% part-time/seasonal and 80% full-time workers, most of whom do not have formal contracts. The owner of Firm 3 explains:

“We cannot afford to employ all full-time workers because our order volume fluctuates seasonally. Having all full-time workers is costly. By reducing the number of full-time workers, we can lower costs and remain profitable, which ensures timely payment for all employees. Additionally, some workers prefer the flexibility of part-time positions, as working in multiple factories allows them to earn more than they would as full-time employees.”

All three small firms complied with 60–70% of health, hygiene, and safety requirements. To achieve full compliance, they are required to expand their floor space, install exhaust fans, transition to energy-efficient light bulbs with higher brightness, and improve ventilation to enhance air quality. The two large firms have fully adhered to all regulations concerning ventilation, spaciousness, access to drinking water, and restroom amenities. They installed air coolers in the factory areas, an optional requirement in the code. At the time of data collection, the small firms were still in rented shared buildings, while the two large firms had moved to their own campuses in industrial areas. These actions improved the working environment in terms of cleanliness and spaciousness and reduced the risk of fire and building collapse for all five firms.

Automation and downsizing unskilled workers offset the increased cost of compliance in firms 1, 2, and 3. As a result, the three small firms decided not to comply with some codes that seemed unnecessary to the owners. For example, while the two large firms had childcare units with pre-schools for the children of workers, the three small firms did not have any childcare facilities. The Chairman of Firm 2 justified this decision:

“Workers feel more secure leaving their children at home with their grandparents rather than bringing them to the factory. Hence, we will try to avoid this cost and use it to provide other, more suitable benefits to the workers. Not sure how long I will be able to do this given the mounting pressure for compliance, but I will try.”

Firm 2 was unable to provide equal promotion opportunities for their female employees and instead opted to have male supervisors on all work floors.

“Due to the presence of high level of masculine cultural values, male leadership is accepted more easily by both male and even female workers. We tried having female supervisors but found that not only male workers, but the female ones also do not take them seriously. Thus, to ensure control and harmony on the factory floor, we decided to have males in supervisory positions.” (Owner of Firm 2)

Suppliers encountered comparable challenges when establishing overtime thresholds. Despite the stipulated limit of 10 h of overtime per week, they could not fully adhere to this standard. This was partly a result of the workers’ preference for overtime and partly because of unexpected orders and unrealistic delivery deadlines from buyers. Because of their poor socio-economic conditions, workers preferred factories that allow overtime. It proved difficult to retain good workers in factories that did not accommodate this preference. Therefore, our sample companies opposed the code to circumvent losing their workforce. The owner of Firm 3 commented:

“One air shipment can squeeze the profit for the entire year. However, we still have to adopt this mode of transportation in order to meet the buyers’ demands, otherwise they may not accept the order and not pay. Last year we had one air shipment before Eid which cost the workers their Eid bonus. Therefore, it is better to deliver on time by practicing overtime than to bear the financial burden which would ultimately put the workers in a worse off situation.”

Another point of compliance pressure pertains to unionization. At the time of data collection, trade unions in Bangladesh were seen by suppliers as extensively politicized and frequently faced allegations of exploiting workers and factory owners to further their own interests (cf. Rahman & Langford, 2021). The alternative solution of the Workers’ Participatory Committee represented a compromise. It provided workers with some freedom of speech; however, it did not support their right to freedom of association. Interviewees justified this with their concerns about violent disruptions and property damage. A Workers’ Participatory Committee had elected representatives and the opportunity to discuss their issues with the management during monthly meetings. The owner of Firm 1 stated:

“In the Bangladeshi context, the trade union has done more harm than good for the workers. Recently, a factory closed due to ongoing labor unrest that was provoked by local union leaders. Now hundreds of workers of that factory are roaming around jobless. Some are even accepting temporary jobs at a negligible hourly rate. So, I believe by preventing my workers from joining the union, I am doing their good as well as protecting my factory from negative influences.”

Besides mitigating measures to protect themselves from the perceived negative outcomes of compliance, supplier firms in this scenario proactively undertook various CSR initiatives that were not on the list of MNE recommendations. As an example, both large firms provide salaries that exceed the legal minimum to enhance the living standards of their employees. Although buyers only stipulate the provision of basic first aid, dining hall, and childcare facilities, both companies offer comprehensive medical services for workers and their families, complimentary lunch and transportation, as well as pre-school education and a parenting resource center for workers’ children. The larger firms went above and beyond this and engaged in dialog with the workers to identify the challenges in their work and personal lives. The next step involved addressing the underlying causes to provide solutions or relief.

“During the 2010–2012 periods, we have started two primary schools with facilities for after school care for our workers’ children in addition to providing them with the mandatory nursery facility [mainly for non-school-age children]. We found that many of our female workers find it difficult to pursue the job as soon as their children reach the school age, with them having to drop and pickup their children to and from school. Some of them chose to continue work without putting their children at school, which we believe would constrain the way for their future generations to get out of the poverty cycle as well... We have interviewed several female workers having children at school age, which enabled us to identify their problem. Hence, we decided to make the school facility available to them at the same compound where they work.” (Owner, Firm 6)

“We have found that several workers switch jobs when their spouse finds a job in a distant place, with them having to move with their spouse. In such a situation, we often recruit the spouse in our factory. In fact, there are incidences that we have employed the entire family, including husband, wife and other young adults in the same family [son, daughter or sibling]. Almost 30% of our workers have their relatives working here. We believe that when the entire family gets to work in a decent place with a decent salary, only then the family can make a way out of poverty. It is also beneficial for our company because when the workers’ entire family is involved in the same factory, they remain more committed and satisfied. This is how we have reduced our turnover rate significantly.” (Compliance Manager, Firm 6)

The three small firms in this group also undertook proactive CSR activities. For example, Firm 1 provided free nutritious lunches and festive bonuses to the workers. Additionally, they engaged in philanthropic activities within the local community during festive occasions and offered regular donations to the local orphanage.

“We provide healthy and balanced lunch to the workers everyday cooked by our company chef. I have seen them [workers] eating only rice and boiled potato for lunch when they used to bring their own food. That is not a balanced diet; it is carbohydrate only. They need protein which they cannot afford to have. Thus, we provided them with free lunch...We also make donations to the community in times of their need. The people in the community expect help from us and we also feel obligated. I personally feel obligated to help workers and the society because of my strong religious values.” (Chairman, Firm 1)

The supplier firms’ proactive CSR activities are motivated by the owners’ values anchored in virtue ethics. Large firms also engage in virtuous activities, albeit on a larger scale. These include the construction of schools for workers and the community, vocational training institutes, free medical treatment, employment opportunities for the workers’ family members, as well as infrastructure development such as roads and electrical substations for the community. For small firms, CSR actions predominantly stem from the virtuous character of the owner/leader, whereas for large firms, they exemplify the virtuous nature of the entire organization. In summary, the findings suggest the supplier firms in this scenario are guided by a combination of consequentialist and virtue ethics.

### Scenario 3: collaborative enhancement

In scenario 3 labeled *collaborative enhancement*, the MNE buyer of Firm 8 stands out in our sample because of their collaborative attitude. The MNE buyer’s decision making was influenced by their internal organizational values, which were clearly stated in their sustainability report.

“Our core products are sourced from a small number of suppliers, enabling us to forge close, long-term relationships.”

“We are committed to building a more transparent supply chain by co-designing our codes to better understand our suppliers’ risks and issues.”

“We believe it is our duty to provide financial and non-financial support to our 1^*st*^* tier suppliers and beyond to help them address their challenges.”*

In their sustainability report, there is evidence that their actions are guided by consequentialist ethical principles and cultural relativism:

“We are conducting a human rights review of our supply chain aligned to United Nations Guiding Principles. We are committed to working together with our suppliers to understand their challenges and thereby derive a mutual solution that generates the optimum outcome for all the stakeholders and are best suited to their socio-economic context.”

Firm 8’s approach to code compliance seems to be strongly guided by their leader’s personal values. The owner of Firm 8 said:

“My commitment to CSR is deeply influenced by my father’s values. He was a devoutly religious man, and a highly respected figure in our village, where my factory is located. He strongly believed in helping the poor, a principle that aligns with the teachings of our religion, Islam, which also advocates for supporting those in need.”

Similarly to supplier firms in the *mitigated forced compliance* scenario, Firm 8’s decisions regarding code compliance were also influenced by consequentialist principles. The two firms collaborated closely to develop and implement the CSR codes. Most of these codes were adapted to the cultural context and put into effect based on cost–benefit evaluations. To illustrate, Firm 8 opted to meet the demands of its workforce by maintaining a workforce composition of 95% full-time and 5% part-time/temporary workers. The MNE buyer supports this arrangement, as they prioritize the workers’ preferences. Firm 8 typically abides by the legal maximum for overtime hours; however, occasionally this limit is exceeded because of extenuating circumstances. Considering that some workers favor overtime for the supplementary income, the MNE buyer does not oppose this practice.

Although the MNE buyer is open to negotiations regarding certain aspects of the codes, such as work arrangements, they maintain strict standards for others, including working conditions and building and fire safety. Firm 8 meets the criteria regarding ventilation and space, offers access to clean drinking water, medical amenities, and maternity benefits. They have built their new factory building in line with the Accord codes for fire and building safety and have achieved a green certificate. The factory floor has a high suction capacity exhaust pipe to control heat and fumes, as well as an air cooler for the comfort of the workers. Unlike other MNEs, the buyer of Firm 8 assists them in meeting these stringent codes by providing both financial and non-financial support.

The code implementation decisions were influenced by the personal and family values of the owner of Firm 8, which emphasize assisting the underprivileged and contributing a share of their wealth to them. Examples include the provision of free medication for employees and their children, as well as the organization of health camps offering free primary care to workers and their immediate family members. These measures were created because of the unavailability of affordable healthcare facilities for workers. Rather than providing a childcare facility of limited value to employees, Firm 8 offered a preschool educational facility in the childcare unit staffed by qualified teachers. This not only promoted the inclusion of workers’ children in the workplace, but also ensured their access to education. Firm 8 also extends interest-free loans to the relatives of its employees to facilitate the establishment of small businesses.

Besides offering prices that exceed the market rate, establishing enduring partnerships, and forecasting annual orders, the multinational enterprise buyer of Firm 8 also shows support for these proactive corporate social responsibility initiatives by providing financial aid for implementing innovative projects. They clearly prioritize meeting the needs of stakeholders and respecting the cultural values of their supplier. Examples include the acceptance of a Workers’ Participatory Committee instead of trade union participation and the engagement of a German consultancy firm to take over the responsibilities of the external trade union. The consultancy firm liaises with the workers and relays their issues to the management. In addition, the consultant provides training to workers to enhance their understanding of their rights and how they can effectively advocate for their rights. To tackle the challenges arising from the prevailing masculine culture and gender roles in Bangladesh, the consultants provide training to male employees on respecting female colleagues and embracing female leadership. Female workers receive training on asserting their rights and managing difficult scenarios as floor supervisors.

The adoption of a collaborative approach, combined with shared ethical values, has not only facilitated code implementation at Firm 8 but has also empowered them to address various issues using culturally suitable methods. As stated by the owner of Firm 8:

“Satisfying the buyers and the worker at the same time is a difficult balancing act. Simply, an understanding attitude of buyers can make such a difference in undertaking the right CSR initiatives. We are lucky because we did not undergo any unnecessary tensions in implementing the Accord codes.”

## Discussion and conclusions

This paper employed a pattern matching approach to investigate the tensions that emerge from differences in the ethical dispositions of MNE buyers and their suppliers within the Bangladeshi apparel manufacturing sector. Our analysis resulted in the identification of four scenarios (see Fig. 1): legitimacy with friction (scenario 1), mitigated forced alignment (scenario 2), and collaborative enhancement (scenario 3). The fourth scenario is conceptual, as none of our sample companies aligned with quadrant 4 of Fig. 1. Here, the supplier firm is guided by deontological principles while the MNE buyer is guided by consequentialist and/or virtue ethics. For the sake of completeness, we labeled it principled resistance.

In Scenario 1 (legitimacy with friction), the expected pattern derived from the literature created the assumption that suppliers will willingly follow MNE codes without considering stakeholders’ immediate contextually anchored needs, because deontological principles like their MNE buyers also guided them. Although the suppliers voiced their appreciation of the MNE buyers’ expertise and acknowledged that the universal principles of these standards will improve working conditions and the competitiveness of the industry, there was a significant difference to our original expectation. There was a hint of consequentialism in their narrative. They followed their buyers’ measures anchored in deontology partly because not doing so would have threatened their firm’s survival. They needed to maintain their relationship with their buyers. As they did not have the resources to mitigate the unintended consequences of code compliance, they had to discontinue previous initiatives targeted at the immediate needs of their workers. The friction in this scenario was produced on two fronts. In their relationship with the MNE buyers, supplier firms felt that their voices were not heard. Our interviewees highlighted that their contextual knowledge could have helped to adapt codes to avoid or mitigate the immediate negative consequences of compliance. In the absence of such dialog and sufficient slack resources to launch countermeasures, tension arose in their relationship with their workers because of the discontinuation of important initiatives. For example, free lunches affecting workers’ ability to meet their nutritional needs.

In Scenario 2 (mitigated forced compliance), supplier firms launched countermeasures. The analysis detected virtue ethics principles in combination with suppliers’ consequentialist stance. Similarly to scenario 1, legitimacy with friction, there was a lack of dialog between MNE buyers and their suppliers which created tensions. Suppliers were questioning their ability to uphold their mitigating measures for an extended period. Thus, in the continued absence of dialog with MNE buyers and MNEs’ willingness to work on just transitions to compliance, we theorize that the mitigated forced compliance would eventually morph into, what Drake and Schlachter (2008) called, a dictatorial collaboration.

In Scenario 3 (collaborative enhancement), we found a match between the ethical disposition of the MNE buyer and the supplier. Both parties displayed a mix of consequentialism and virtue ethics. The MNE buyer showed an understanding of contextual challenges and a willingness to work with the supplier to find alternative solutions that were mutually acceptable. The buyer also supported their supplier’s code implementation with financial and non-financial contributions. This scenario comes close to what Drake and Schlachter (2008) describe as sustainable collaboration.

In the conceptual Scenario 4 (principled resistance), the MNE buyer would be guided by consequentialist and/or virtue ethics principles, while the supplier would follow deontology. It needs to be reiterated that this matrix emerged from the data analysis, thus we did not explicitly seek firms that would fit this scenario in our theoretical sampling process. We theorize that suppliers in this scenario would possess adequate leverage to resist the demands of their multinational enterprise buyers without risking their business relationship. Suppliers would insist on only complying with the requirements outlined in a standard or code and not be willing to consider mitigating undesirable or unintended consequences. Alternatively, in such a scenario, suppliers might have their own standards or codes that differ from Western standards and be powerful enough to refuse to deviate from them. Future research is needed to explore these conjectures empirically.

Out of the four scenarios in Fig. 1, scenario 3 (collaborative enhancement) seems to be the most suited to achieve a just transition to a more desirable state within a context that faces development challenges and institutional gaps. This is in line with van Tulder and van Mil’s (2022) assertion that to address wicked problems, a combination of ethical principles is needed, because wicked problems have no stopping rule. Suppliers can be an important source of knowledge for MNEs in the mapping of binding and non-binding constraints (Sinkovics et al., 2014) that are connected to wicked problems. Naudé (2011:p. 34) defines binding constraints as “circumstances or factors which, as long as they remain in place, would hinder [economic] growth, even if other possible constraints or determinants of growth are addressed.” As a result, binding constraints can only be addressed at a macro level and require cross-sectoral partnerships, often at a transnational level. Non-binding constraints are circumstances or factors that hinder the sustainability/well-being of a business and/or that of its stakeholders (cf. Sinkovics et al., 2014). Building on this thinking, Sinkovics et al. (2015) define social constraints as the root causes that keep systems from attaining their socially oriented goals. How systems are defined depends on how we set their boundaries. In our context, we can define several interconnected systems that would correspond to stakeholder groups, i.e., workers, suppliers, wider community, etc. Interventions targeted at root causes or significant symptoms of these root causes require ongoing feedback, adaptation to the context, continuous refinement, and active dialog with stakeholders (e.g., Sinkovics et al., 2021; van Tulder & van Mil, 2022).

However, despite their resource richness, MNEs have a disadvantage in understanding the root causes of systems in geographically and psychically distant locations, partly because of their liability of outsidership and liability of foreignness (Sinkovics et al., 2014). Reducing the liability of outsidership and foreignness is already time and resource intensive in the business domain (Johanson & Vahlne, 2009; Yamin & Kurt, 2018), adding the social domain to the mix with its cultural relativism and potential institutional voids adds a further layer of complexity and requires a different set of capabilities. To overcome these liabilities, MNEs need to engage in a process of interactive learning and the development of relationships with relevant host country organizations (Yamin & Kurt, 2018). From a GVC analysis perspective, the willingness of MNEs to engage in such a resource intensive interactive learning process depends on the complexity of transactions, the codifiability of the transactions, and suppliers’ level of capability to deliver the buyers’ requirements (Gereffi et al., 2005). MNEs will be less inclined to listen to the voice of supplier unless they directly derive benefits from frequent two-way communication or ensuring compliance without such resource intensive and interactive mechanisms is not possible (cf. Cano-Kollmann et al., 2016; Ponte & Sturgeon, 2014). At face value, opting to be guided by deontological principles taking the shape of standards and codes may seem to increase efficiency and reduce the cost of coordination, especially when MNE buyers are in a powerful position vis-à-vis their suppliers.

Therefore, although the effectiveness of standards as governance mechanism has been increasingly questioned (e.g., Lee et al., 2019; Locke, 2013; Lund-Thomsen, 2008; Sinkovics et al., 2016; Van Assche & Narula, 2022), without significant external or internal incentives, MNEs are not likely to prioritize the effectiveness of CSR/labor standards and codes over their efficiency. In the domain of marketing strategy standardization and adaptation, empirical results show that a strategy only leads to superior performance when lead firms achieve a sufficient level of co-alignment between the implemented strategy and key contextual factors (Katsikeas et al., 2006). In the context of CSR/labor standards and codes, the emphasis should be on the performance of the standards/codes in terms of meaningful development impact. If MNEs’ strategic goals are directed by deontological instead of virtue ethics or a combination of consequentialist and virtue ethics principles, the development outcome of the governance mechanisms will be suboptimal. In order for MNEs to “bear their fair share of responsibility for finding solutions to prevailing human rights problems” (Wettstein, 2012: p. 753) and to other grand challenges outlined in the sustainable development goals (SDGs) (cf. Buckley et al., 2017), there needs to be a change to what are acceptable ethical guiding principles at the C-suite level (cf. Elkington & Braun, 2013).

Further, the dogma of the ethical neutrality as opposed to ethical relativism in setting developmental goals and operationalization strategies has been a point of contestation for development theorists-practitioners (Crocker, 1991, 2008; Keleher & Kosko, 2019). Crocker (2008) suggests academics and practitioners in development studies forge ethically based but operational criteria for what counts as development success and failure. He advocates for having *“a global dialogue that occurs in a context in which the big, strong, and rich do not coerce the small, weak, and poor (*Crocker, 1991*: p. 474).”* Drawing on normative ethical theories, development ethicist Keleher (2019) highlights the importance of avoiding a one-size-fits-all strategy for guiding moral behavior, while also cautioning against relying solely on complete moral relativism as it leaves the door open to exploitation. Therefore, he suggests exploring the credibility of a middle ground as a suitable development ethics.

By delving into the supplier’s perspective, our findings add further nuances to understanding the unintended consequences of pushing standards down the chain with no room for adaptations to allow just transitions. These insights can help MNEs explore the intention and realization gap of their interventions (van Tulder & van Mil, 2022). Most importantly, our findings strengthen the argument that supplier voice should factor in more prominently in the design and implementation of standards and codes (Bennett, 2017; Hoque et al., 2016; Khan & Lund-Thomsen, 2011) if their effectiveness is to be improved. Future research will need to collect dyadic large-scale data to expand on our insights to generate and test hypotheses across different industries and contexts.

## Data Availability

The data supporting the findings of this study are not publicly available due to the sensitive nature of the information. Access to the data is restricted to protect confidentiality, privacy, or other ethical considerations.
